# Impairment of quality of life in parents of children and adolescents with pervasive developmental disorder

**DOI:** 10.1186/1477-7525-5-22

**Published:** 2007-04-27

**Authors:** Diego Mugno, Liliana Ruta, Valentina Genitori D'Arrigo, Luigi Mazzone

**Affiliations:** 1 Division of Child Neurology and Psychiatry, Department of Pediatrics, University of Catania, Via S. Sofia 78, 95100 Catania, Italy

## Abstract

**Background:**

Little is known about the Quality of Life (QOL) in parents of children with developmental diseases as compared to other severe neurological or psychiatric disorders. Aims of the present study were: to evaluate QOL in parents of children affected by Pervasive Development Disorder (PDDs), Cerebral Palsy (CP) or Mental Retardation (MR) as compared to a control group (CG); to evaluate QOL of parents of patients with different types of PDDs, namely Autistic Disorder (AD), High Function Autism/Asperger Syndromes (HFA/AS) and Pervasive Developmental Disorder Not Otherwise Specified (PPD-NOS); and to compare the level of impairment in QOL of mothers and fathers within PDDs, CP, MR groups and between AD, HFA/AS, PDD-NOS sub-groups.

**Methods:**

The sample consisted of 212 parents (115 mothers and 97 fathers) of 135 children or adolescents affected by PDDs, MR or CP. An additional sample of 77 parents (42 mothers and 35 fathers) of 48 healthy children was also included and used as a control group. QOL was assessed by the WHOQOL-BREF questionnaire.

**Results:**

Compared with parents of healthy children, parents in the PDDs group reported impairment in physical activity (p = 0.0001) and social relationships (p = 0.0001) and worse overall perception of their QOL (p = 0.0001) and health (p = 0.005). Scores in the physical (p = 0.0001), psychological (p = 0.0001) and social relationships domains (p = 0.0001) and in the physical (p = 0.0001) and social relationships (p = 0.0001) domains were lower compared to the MR group CP group respectively. Little differences were observed between MR, CP and control groups. The level of impairment of physical (p = 0.001) and psychological (p = 0.03) well-being were higher in mothers than in fathers in the PDDs and CP groups respectively; in the other groups, and across all the other domains of QQL impairment was similar. There were no statistically significant differences in the scores between the AD, HFA/AS and PDD-NOS sub-groups, but parents in the HFA/AS sub-group seemed to display a lower QOL compared to the AD sub-group.

**Conclusion:**

Parents of children with PDDs seem to display a higher burden, probably for a combination of environmental and genetic factors. Within this group of parents also those of HFA or AS people have higher stress. These finding must be taken into account in policy making to provide better and more specific supports and interventions for this group of diseases.

## Background

Quality of Life (QOL) has been defined by the World Health Organization as individuals' perception of their position in life in the context of the culture and value systems in which they live and in relation to their goals, expectations, standards and concerns. It is a broad concept incorporating the person's physical health, psychological state, level of independence, social relationships, personal beliefs and their relationship to salient features of the environment [1]. As such, QOL cannot be simply equated with the terms health status, life style, life satisfaction, mental state, or well-being; rather, it is a multidimensional concept incorporating the individual's perception of these and other aspects of life [2].

Assessment of QOL is important in medical practice, to improve the doctor-patient relationship, in assessing the effectiveness and relative merits of different treatments, in health services evaluation, in research and in policy making [3].

QOL is especially relevant to conditions that are chronic and impairing, such as pervasive developmental disorder (PDD), Cerebral palsy (CP), mental retardation (MR).

PDDs, CP and MR are not rare conditions in the population. Prevalence of PPD has been estimated to be around 1–5 per 1,000 [4,5] and an increase in incidence of this disease has been reported by clinicians, schools, and service agencies worldwide [6,7] Prevalence of MR is between 3–7 subjects per 1,000 [8,9] and for CP have been reported to be between 1.5–3 per 1000 children [10].

Parents of children with developmental disabilities experience heightened stress, [11,17], impaired mental health [18], sense of devaluation and self-blame [19], impaired physical functioning, tiredness or exhaustion [20,21]. The level of Impairment in quality of life within families of children with these severe chronic conditions is likely to be moderated by a complex matrix of environmental as well as genetically-based variables such as socio-economic status, social support, parental and child characteristics and coping strategies [22,23]. The level of parental stress has been found to be related to the level of severity and disability of the children's diagnoses and to coexisting behavioral problems [24,25]. Mothers of children with PDDs reported higher levels of stress and demoralization than fathers [26,27]. Research has indicated that maternal stress in families with children with autism [28,29] is predicted by their children's co-existent behaviour problems and also by their partner's depression. It has been suggested that co-existent behaviour problems in the child predict parental stress to a higher extent than the severity of the autistic symptoms [27-29]. Furthermore mothers of children with severe mental disorder and with intellectual disability showed increased rates of physical health problems with poor perception of their health [24,30,31].

Little data are available on the QOL in parents of children with developmental diseases as compared to other severe neurological or psychiatric disorders. We assessed QOL of parents of children and adolescent affected by pervasive developmental disorders (PDDs), cerebral palsy (CP), mental retardation (MR) and normally developing (control group, CG).

Aims of this study were: 1) to evaluate QOL in parents of children suffering from PDDs, CP or MR as compared to a control group; 2) to evaluate QOL of parents of patients with different types of PDDs, namely Autistic Disorder (AD), High Function Autism/Asperger Syndromes (HFA/AS) and Pervasive Developmental Disorder not otherwise specified (PPD-NOS); 3) to compare the level of impairment in QOL of mothers and fathers within PDDs, CP, MR groups and between AD, HFA/AS, PDD-NOS sub-groups. We hypothesized that parents of children of PPD might display an higher impairment of quality of life as compared to the other groups because patients with PPD usually display more and/or more severe maladaptive behaviors and have lower chance of achieving a better psychological adjustment compared to people with CP and MR. The presence of genetic liability for autism (broad autism phenotype) in family of autistic probands as well as the higher aggregation of psychiatric disorders in these families will be take into account.

## Methods

### Sample

The recruited sample comprised parents of 160 children or adolescent affected by PDDs, MR or CP (60 children or adolescent affected by PDDs; 60 affected by MR; 40 affected by CP). For each subject 2 questionnaires were provided to parents independently of the family status, for a total number of 320 questionnaires (120 questionnaires for the PDDs group, 120 questionnaires for the MR group, 80 questionnaires for the CP group).

An additional sample of parents of 65 healthy children was also included and used as a control group (CG), for a total number of 130 questionnaires.

### Assessment of QOL

The World Health Organization (WHO), with the aid of 40 collaborating centers around the world, has developed two self-administered instruments for measuring quality of life (the WHOQOL-100 and the WHOQOL-BREF), that can be used in a variety of cultural settings whilst allowing the results from different populations and countries to be compared.

The WHOQOL-BREF contains two items from the Overall Quality of Life and General Health, and one item from each of the 24 facets included in The WHOQOL-100. Analysis of The WHOQOL-100 structure has suggested the possibility of merging two of six domains of the WHOQOL-100, thereby creating four domains of quality of life: physical, psychological, social relationships and environment [32].

The WHOQOL-BREF produces a profile with four domain scores and two individually scored items about an individual's overall perception of QOL and health (Q1 and Q2). The four domain scores are scaled in a positive direction, with a score range of 0–100, and with higher scores denoting higher QOL. The two individual items assessing overall QOL are scaled in a positive direction, with a score range of 1–5 (converted in this study into a 0–100 score), with higher scores denoting higher QOL.

Psychometric properties of the Italian version of the WHOQOL-Bref have been tested by the "*Centro Italiano Collaborativo Progetto WHOQOL" *[33].

### Procedure

This study was conducted at the Department of Child Neurology and Psychiatry of the University of Catania, Italy, Participants were parents of people aged 2–18 years referred to the center during a period of 1 year (June 2005–June 2006).

The participating children underwent a comprehensive clinical diagnostic assessment based on the DSM-IV-TR criteria [34], and all diagnoses were made according to DSM-IV-TR criteria except when specified.

Potential participants could enter the study if their sons received a diagnosis of PPDs, MR or CP.

The CG consisted of mothers and fathers of typically developing people aged <18, recruited via school nurses, attending regular classes in mainstream schools, with no mental, developmental, or physical disabilities according to school medical records and not receiving ongoing prescription medication.

Exclusion criteria were inability to undergo the written parts of the questionnaire or refusal to participate. Parents of more than 1 son affected by PDDs, MR or CP were not included in the study. Questionnaires missing more than 20% of data, missing items Q1 or Q2 or missing more than two items from the domain (more than 1 for domain 3, social relationships), were discarded. Where an item was missing, the mean of other items in the domain was substituted [3].

The instrument used to assess parental QOL was distributed by the authors to the families of the study group during inpatient or outpatient visits, during home visit or by mail and email and were returned to the authors via a parental visit to the clinic, a second home visit or by mail and email. The questionnaire was distributed to the families of the CG by mail. Each parent separately filled in the QOL instrument. The questionnaires were anonymous. The cover page, gave information about the study, brief instructions and an example of how to respond to the questions. Demographic and health questions are not included in the WHOQOL-BREF and were included as a separate partially structured demographic questionnaire. The requested data were gender, age, family status, school education and health status of parents; age and gender of children. Data from the demographic questionnaire were only used to assess statistical differences between the groups on the requested information.

### Data analysis

Data were assessed using Student's *t*-tests and one-way ANOVAs with post hoc Scheffé multiple comparison tests. The Statistical Package for Social Sciences (SPSS) [35] was used. Significance level *p *< .05 was regarded as statistically significant.

## Results

Among the PDDs group, 51 questionnaires were not included in the study, constituting a drop-out rate of 42.5%. 39 of the 51 excluded questionnaires (76%) were not returned to the authors; 12 of the 51 excluded questionnaires (24%) were considered ineligible for the analysis because of missing data.

Among the MR group, 31 questionnaires were excluded from the study, constituting a drop-out rate of 25.8%. 19 of the 31 excluded questionnaires (62%) were not returned to the authors; 12 of the 31 questionnaires (38%) were excluded for missing data.

In the CP group, 26 questionnaires were not included in the study, constituting a drop-out rate of 32.5%. 19 questionnaires (73%) were not returned to the authors; 7 questionnaires (27%) were considered ineligible for the analysis because of missing data.

A total number of 289 questionnaires were included in the study.

The study group consisted of 212 parents (97 fathers, 115 mothers) of 135 children or adolescents affected by PDDs, MR or CP. The control group (CG) consisted of 77 parents (35 fathers and 42 mothers) of 89 healthy children.

The PDDs group consisted of 69 parents (30 fathers and 39 mothers) of 53 children and adolescent (males n. 42; females n. 11; sex ratio M/F = 3.8/1) affected by PDDs. 16 fathers and 21 mothers of 26 children and adolescents (males n. 20; females n. 6) affected by autistic disorder (AD) and IQ level<70 (n. 16, IQ between 55 and 70; n. 10, IQ between 40 and 54), 10 fathers and 12 mothers of 20 children and adolescents (males n. 17; females n. 3) affected by Asperger's disorder or high-functioning autism (HFA/AS), the latter defined as AD with IQ level>70, 4 fathers and 6 mothers of 7 children and adolescents (males n. 5; females n. 2) affected by Pervasive Developmental Disorder-Not otherwise specified (PDD-NOS) (n. 3, IQ between 55 and 69; n. 4, IQ between 40 and 54).

The MR group consisted of 89 parents (40 fathers and 49 mothers) of 55 children and adolescents (males n. 33; females n. 22; sex ratio M/F = 1.5/1) affected by MR without autism spectrum conditions. 23 fathers and 28 mothers of 32 children and adolescents (males n. 18; females n. 14) affected by mild to moderate MR, 17 fathers and 21 mothers of 23 children and adolescents (males n. 15; females n. 8) affected by severe to profound MR.

The CP group consisted of 54 parents (27 fathers and 27 mothers) of 30 children and adolescents (males n. 15; females n. 15; sex ratio M/F = 1/1) affected by CP. For this group we have adopted the classification of subtypes of cerebral palsy agreed by the Surveillance of Cerebral Palsy in Europe collaboration of cerebral palsy registers [36]. 12 fathers and 13 mothers of 12 children and adolescent (males n. 7; females n. 5) affected by 2 limbs spastic bilateral type CP (2SBCP), 9 fathers and 7 mothers of 10 children and adolescents (males n. 4; females n. 6) affected by hemiplegia type CP (HCP), 6 father and 7 mother of 8 children and adolescents (males n. 4; females n. 4) affected by 3–4 limbs spastic bilateral type CP (SBCP).

The CG consisted of 42 mothers and 35 fathers of 48 typically developing children (males n. 17; females n. 31; sex ratio M/F = 1/1.8) recruited via school nurses, attending regular classes in mainstream schools, with no mental, developmental, or physical disabilities according to school medical records and not receiving ongoing prescription medication.

Average age of parents was 40 ± 13.5 years (20–58 years). Family status was: 76% married/cohabitating, 21% separated/divorced, 3% widowed. Level of education was: 39% primary school, 44% secondary school, 17% university. None of the parents was currently ill.

Average age of children within PDDs group was 7.5 ± 5 years (range 3–17). Average age of children within the MR group was 6.3 ± 7 years (range 4–16). Average age of children within the CP group was 9 ± 5 years (range 2–16). Average age of children within the CG was 8 ± 4 years (range 4–15). There were no statistically significant differences regarding socio-demographic factors between the groups (Table 1).

**Table 1:** Demographics characteristics of parents of children and adolescents with Pervasive development disorder (PDDs), Mental retardation (MR), Cerebral Palsy (CP) and controls CG).

	PDDs (N = 69)	MR (N = 89)	CP (N = 54)	CG (N = 77)	TOT (N = 289)
Fathers/Mothers	30/39	40/49	27/27	35/42	132/157
Age (Mean ± SD)	37 ± 12.7	43 ± 14.5	39 ± 12.5	41 ± 14.3	40 ± 13.5
Family status					
Married/Cohabitating	53 (77%)	67 (75%)	40 (74%)	60 (68%)	220 (76%)
Separated	15 (22%)	18 (20%)	12 (22%)	16 (21%)	61 (21%)
Widowed	3 (4%)	2 (2%)	2 (4%)	2 (3%)	9 (3%)
Level of Education					
Primary	26 (38%)	35 (39%)	22 (41%)	30 (39%)	113 (39%)
Secondary	30 (43%)	39 (44%)	24 (44%)	34 (44%)	127 (44%)
University	12 (17%)	14 (16%)	10 (19%)	13 (17%)	49 (17%)

Sons/Daughters	42/11	33/22	15/15	17/31	107/79
Age (Mean ± SD)	7.5 ± 5	6.3 ± 7	9 ± 5	8 ± 4	7.7 ± 5.2

### Comparison between the PDDs, the MR, the CP and the control groups

Fathers in the PDDs group showed statistically significant lower scores in the social relationship domain and in the Q1 item compared to CG, and in the psychological domain compared to CP group (Table 2). Mothers in the PDDs group showed statistically significant lower scores in the Q1 and Q2 items and in the physical and social relationship domains compared to the CG, and in the psychological domain compared to the MR group. Mothers in the MR group showed statistically significant lower scores Q1 item and in the physical domain compared to the CG. Mothers in CP group showed statistically significant lower scores in the Q1 item compared to the CG (Table 2).

**Table 2:** Comparison of Quality of Life (WHOQOL-BREF) between fathers and mothers of children and adolescents with Pervasive development disorder (PDDs), Mental retardation (MR), Cerebral Palsy (CP) and controls (CG).

**Fathers**	PDDs (N = 30)	MR (N = 40)	CP (N = 17)	CG (N = 35)	ANOVA		Post-hoc contrasts
					F	p	
Q1 (mean ± SD)					7.28	0.000	CG>PDDs
Q2 (mean ± SD)	65.83 ± 19.12	72.16 ± 18.06	63.24 ± 20.00	74.29 ± 22.27	1.8	0.151	
Physical (mean ± SD)	65.48 ± 11.11	67.37 ± 12.83	64.92 ± 17.05	72.24 ± 14.80	1.77	0.156	
Psychological (mean ± SD)	64.58 ± 16.00	71.69 ± 12.41	76.47 ± 9.65	68.93 ± 14.93	3.05	0.031	CP>PDDs
Relationships (mean ± SD)	60.00 ± 18.36	68.37 ± 16.13	72.06 ± 9.29	75.24 ± 15.59	5.27	0.002	CG>PDDs
Environment (mean ± SD)	53.75 ± 12.70	55.61 ± 12.46	59.01 ± 12.59	56.96 ± 17.78	0.56	0.640	
**Mothers**	PDDs (N = 39)	MR (N = 49)	CP (N = 27)	CG (N = 42)	F	p	

Q1 (mean ± SD)	58.33 ± 21.71	63.27 ± 20.48	60.35 ± 16.53	77.98 ± 13.75	9.16	0.000	CG>PDDs, MR, CP
Q2 (mean ± SD)	55.77 ± 26.57	67.35 ± 21.77	65.29 ± 19.98	71.43 ± 21.08	3.47	0.018	CG>PDDs
Physical (mean ± SD)	53.94 ± 16.34	64.65 ± 15.91	60.87 ± 12.99	68.45 ± 15.68	6.44	0.000	CG>PDDs, MR
Psychological (mean ± SD)	57.59 ± 17.41	68.79 ± 13.40	67.20 ± 15.68	64.38 ± 15.40	4.17	0.007	MR>PDDs
Relationships (mean ± SD)	58.97 ± 23.68	69.73 ± 16.47	66.97 ± 15.99	72.22 ± 18.56	3.72	0.013	CG>PDDs
Environment (mean ± SD)	48.96 ± 14.84	57.14 ± 11.71	54.64 ± 12.60	54.24 ± 19.00	2.24	0.086	

Q1 = Overall perception of Quality of Life; Q2 = Overall perception of Health.

### Comparison between fathers and mothers within the PDDs, the MR, the CP and control groups

Within the PDDs group mothers showed lower scores in the physical domain (p = 0.001) and within the CP group in the psychological domain (p = 0.03). No statistically significant differences were observed within the MR group and the CG (Table 3).

**Table 3:** Comparison of Quality of Life (WHOQOL-BREF) between fathers and mothers of participants with Pervasive Development Disorder (PDDs). Mental Retardation (MR). Cerebral Palsy (CP) and control group (CG).

	**PDDs fathers n.30**	**PDDs mothers n.39**		**MR fathers n.40**	**MR mothers n.49**		**CP fathers n.27**	**CP mothers n.27**		**Control fathers n.35**	**Control Mothers n.42**	
	Mean (SD)	Mean (SD)	***p***	Mean (SD)	Mean (SD)	***p***	Mean (SD)	Mean (SD)	***p***	Mean (SD)	Mean (SD)	***p***

Q1	59.17 (16.72)	58.33 (21.71)	N.S.	67.05 (17.7)	63.27 (20.48)	N.S.	66.18 (17.55)	60.35 (16.53)	N.S.	78.57 (16.21)	77.98 (13.75)	N.S.
Q2	65.83 (19.12)	55.77 (26.57)	N.S.	72.16 (18.06)	67.35 (21.77)	N.S.	63.24 (20)	65.29 (19.98)	N.S.	74.29 (22.27)	71.43 (21.08)	N.S.
Physical	65.48 (11.11)	53.94 (16.34)	0.001	67.37 (12.83)	64.65 (15.91)	N.S.	64.92 (17.05)	60.87 (12.99)	N.S.	72.24 (14.8)	68.45 (15.68)	N.S.
Psychological	64.58 (16)	57.59 (17.41)	N.S.	71.69 (12.41)	68.79 (13.4)	N.S.	76.47 (9.65)	67.2 (15.68)	0.03	68.93 (14.93)	64.38 (15.4)	N.S.
Relationships	60 (18.36)	58.97 (23.68)	N.S.	68.37 (16.13)	69.73 (16.47)	N.S.	72.06 (9.26)	66.97 (15.99)	N.S.	75.24 (15.59)	72.22 (18.56)	N.S.
Environment	53.75 (12.7)	48.96 (14.84)	N.S.	55.61 (12.46)	57.14 (11.71)	N.S.	59.01 (12.59)	54.64 (12.6)	N.S.	56.96 (17.78)	54.24 (19)	N.S.

### Comparison within the PDDs group: the AD, HFA/AS and PDD-NOS sub-groups

Fathers in the AD sub-group showed statistically significant lower scores in the Q1 item and in the social relationship domain compared to CG. Fathers in the HFA/AS sub-group showed statistically significant lower scores in the Q1 and Q2 items and in the social relationship domain compared to CG, and in the Q2 item compared to the PDD-NOS sub-group (Table 4). Mothers in the AD sub-group showed statistically significant lower scores in the Q1 item and in the physical domain compared to CG. Mothers in the HFA/AS sub-group showed statistically significant lower scores in the Q1 item and in the physical and social relationship domains compared to CG. Mothers in the PDD-NOS sub-group showed statistically significant lower scores Q1 item compared to CG (Table 4).

**Table 4:** Comparison of Quality of Life (WHOQOL-BREF) between fathers and mothers of children and adolescents with Autistic Disorder (AD), Asperger's disorder or high-functioning autism (HFA/AS) and Pervasive Developmental Disorder-Not otherwise specified (PDD-NOS).

**Fathers**	AD (N = 16)	HFA/AS (N = 10)	PDD-NOS (N = 4)	CG (N = 35)	ANOVA		Post-hoc contrasts
					F	p	
Q1 (mean ± SD)	56.25 ± 19.3	60.00 ± 12.9	68.75 ± 12.5	78.57 ± 16.21	8.11	0.000	CG>AD, HFA/AS
Q2 (mean ± SD)	68.75 ± 14.43	52.50 ± 18.45	87.50 ± 14.43	74.29 ± 22.27	4.25	0.009	CG>HFA/AS; PDD-NOS>HFA/AS
Physical (mean ± SD)	65.40 ± 11.04	64.64 ± 12.65	67.86 ± 9.67	72.24 ± 14.80	1.42	0.245	
Psychological (mean ± SD)	62.24 ± 16.14	63.33 ± 16.64	77.08 ± 9.92	68.93 ± 14.93	1.47	0.231	
Relationships (mean ± SD)	59.90 ± 14.97	56.67 ± 23.17	68.75 ± 19.69	75.24 ± 15.59	4.81	0.005	CG>AD, HFA/AS
Environment (mean ± SD)	53.91 ± 14.05	50.94 ± 11.70	60.16 ± 8.98	56.96 ± 17.78	0.55	0.651	
**Mothers**	AD (N = 21)	HFA/AS (N = 12)	PDD-NOS (N = 6)	CG (N = 42)	F	p	

Q1 (mean ± SD)	58.33 ± 19.90	60.42 ± 22.51	54.17 ± 29.23	77.98 ± 13.75	8.01	0.000	CG>AD, HFA/AS, PDD-NOS
Q2 (mean ± SD)	54.76 ± 26.95	56.25 ± 28.45	58.33 ± 25.82	71.43 ± 21.08	2.87	0.042	
Physical (mean ± SD)	53.23 ± 17.42	53.57 ± 16.19	57.14 ± 14.98	68.45 ± 15.68	5.52	0.002	CG>AD, HFA/AS
Psychological (mean ± SD)	58.73 ± 16.66	51.74 ± 19.82	65.28 ± 13.09	64.38 ± 15.40	2.17	0.099	
Relationships (mean ± SD)	60.71 ± 22.38	52.08 ± 21.94	66.67 ± 31.62	72.22 ± 18.56	3.38	0.022	CG>HFA/AS
Environment (mean ± SD)	48.96 ± 15.76	44.79 ± 11.02	57.29 ± 16.96	54.24 ± 19.00	1.35	0.263	

Q1 = Overall perception of Quality of Life; Q2 = Overall perception of Health.

### Comparison between fathers and mothers within the AD, HFA/AS, PDD-NOS

The only statistically significant differences was the lower scores of mothers in the physical domain within the AD sub-group (p = 0.02).

## Discussion

Parents of children with PDDs showed a significant impairment of QOL as compared to the other groups, while little differences were observed between MR, CP and control groups. In particular, significant differences in the MR and CP groups compared to controls where present only in mothers, with impairment in the physical domain and overall perception of QOL in mothers of children with MR and in overall perception of QOL for mothers of children affected by CP. No statistically significant differences were observed between MR and CP groups.

Within the PDDs group mothers tended to have a lower QOL compared to fathers, despite the only statistically significant difference found between fathers and mothers was in the physical domain (mothers of children with PDDs showing lower scores). More specifically, mothers of children with PDDs displayed lower physical health, impairment in social relationship and in the psychological state, and a worse overall perception of QOL and health, while fathers displayed a worse perception of their psychological state and impairment in overall QOL and in social relationship.

These findings are in accordance with previous studies, reporting parents of children with autism, particularly mothers, experience more stress than parents of typically developing children or other clinical conditions (cystic fibrosis, Down syndrome, behaviour disorders, mental retardation, learning-disability) [12,19,27,37,45].

Analyzing data within the PDDs group, the HFA/AS sub-group seems to display a lower QOL compared to the AD sub-group, because of the lower scores in more domains. In particular, the fathers' HFA/AS sub-group shows impairment in overall perception of QOL and health (the latter also compared to the PDD-NOS subgroup) and in the social relationship domain, while the fathers' AD sub-group only in overall perception of QOL and in social relationship; the mothers' HFA/AS sub-group displays impairment in overall perception of QOL, in psychical health and in social relationships while mothers' AD sub-group only in overall perception of QOL and physical health.

From an environmental stand-point, the differences in QOL between parents of children with PDDs and parents of children with other clinical diagnoses could be attributed to the environmental effects (greater stresses and burden) of having a child with such severe developmental disorders: difficult behaviors, including temper tantrum and aggressive, self-abusive, destructive, obsessive, ritualistic, impulsive and self-stimulatory behaviors; limited social skills and judgment that often resulted in being teased or rejected; the strain of not understanding their children or knowing what was wrong with them; needed constant supervision and assistance with daily living skills; financial strains; the problems associated with school and relative services; difficulty obtaining a correct diagnosis; stressful experiences with professionals; worries about the future, including living arrangements and sexuality; ineffective services and unmet needs; poor communication and coordination among services providers [46-48].

On the other hand, studies undertaken from the late 1970s indicated the presence of strong genetic influences and of a phenotype much broader than the traditional diagnostic category of autism [49]: twin and family studies [50], as well as observations on the familiality of a range of traits (social, communication and language difficulties, personality traits, vocational interests, cognitive style) linked to autism [51-63] provide direct and indirect evidences for the existence of a genetic contribution and of a genetic liability for autism spectrum disorders [64]: 'the lesser variant' [65] or 'the broad autism phenotype" [62,66].

Other studies report familial aggregation of psychiatric disorders in families of autistic individuals: obsessive-compulsive disorder, tic disorders, affective disorders (especially major depressive disorder), anxiety disorders (in particular social phobia) and personality disorders [67,68].

A few reports of psychological tests (Minnesota Multiphasic Personality Inventory, Eysenck Personality Inventory, Maudsley Personality Inventory) administered to parents of children with autism in the 1970' and 1980' indicated no significant finding [40,69,70]. However a variety of studies using self-report measures of various symptoms of depression and anxiety found increased reports of psychological distress in parents of children with autism (especially mothers) compared to parents of typical children, those with Down syndrome, or other clinical groups [15,45,71-73]. Further, researchers have recently documented increased rates among parents of children with autism of clinical depression compared to parents of children with Down syndrome [61-63]; but these studies have documented in the majority of cases of major depression, episodes of the disorder had occurred before the birth of the child with autism. The same studies also found increased rates in parents of children with autism of social phobia and other anxiety disorders and some indication of increased incidence of obsessive-compulsive disorder in the extended families.

Piven and Palmer (1999) [74] has suggested two main hypothesis to explain the finding of increased rate of depression among parents of children with autism: 1. a genetic predisposition to depression in individuals who later produce a child with autism or 2. individuals who are depressed or anxious have an increased incidence of marring/having children with a partner who is genetically predispose to produce a child with autism.

Depression seems to be associated with depression in other family members but not with the broader phenotype and with the severity of autism; its increased rate in the families of individuals with autism  has not been yet explained but it does not seem to reflect a genetic liability to autism [49].

Another not clarified point is if parents of HFA or AS sons display higher stress compared to parents of children with PDD and mental retardation [75-77].

In our study, parents of children with PDDs showed a significant impairment of QOL as compared to the other groups, and parents in the HFA/AS sub-group seemed to display a lower QOL compared to the AD sub-group.

At the moment it's difficult to examine genetic and environmental influences independently.

The highest impairment of QOL that we found in parents of children with PDDs, might be the expression of this underlying genetic predisposition (both the liability to autism and the higher familiar rate of psychiatric disorders) combined with environmental precipitants.

Further researches are needed to better explain the complicated genes-environment interaction.

Results of the present study should be considered in the context of the following limitations:(1) we did not take into account important factors that might influence QOL such as the socioeconomic status of the parents; in particular no correlational or multivariate analyses with regard to predictors or determinants of parental QoL have been performed; (2) we did not assessed psychiatric comorbidity in the children; (3) this study did not evaluate the options of treatment for the children; (4) the limited number of the study group probably makes the sample not totally representative for the population of parents of children affected by PDDs, MR or CP; (5) the wide age range of the participants, may be another limitation, despite research results have been mixed as to the effect of the child's age on parental distress [37,39,71,72,74]. Some studies support the idea that mothers tend to experience distress earlier than fathers do, perhaps as a combination of childcare demands and early awareness of the child's impairments [37,39]; (6) age of diagnosis and gender of the children were not taken into consideration and no statistical analyses regarding these factors have been performed; (7) the instrument used to assess parental QOL was distributed by the authors to the families of the study group during inpatient or outpatient visits, during home visit or by mail and email and were returned to the authors via a parental visit to the clinic, a second home visit or by mail and email. The questionnaire was distributed to the families of the CG by mail. This difference in assessment methods may have contributed in an unknown way to the differences in the QoL scores between the groups.

## Conclusion

Parents of children with PDDs seem to display a higher burden, probably for a combination of environmental and genetic factors. Within this group of parents also those of HFA or AS people have high stress. These finding must be taken into account in policy making to provide better and more specific supports and interventions for this group of diseases.

More attention should be given to parents' (and in particular mothers') needs. Social support and different coping strategies should be developed to respond positively to individual changing needs and in buffering parents from the stress of having a child with disability [15,44,45,78-80]. New research should be conducted to measure the effectiveness of these strategies. In addition, effective and sustainable psycho-social programs are needed to provide necessary support for the special needs of the children and their families.

## Competing interests

The author(s) declare that they have no competing interests.

## Authors' contributions

DM designed the study, collected the data, performed statistical analysis and drafted the manuscript

LR and VGD assisted in design of the study and commented on the draft paper

LM designed the study, drafted the manuscript together with DM and participated in the interpretation of data

All authors read and approved the final manuscript.
